# Adaptive design clinical trials: Methodology, challenges and prospect

**DOI:** 10.4103/0253-7613.68417

**Published:** 2010-08

**Authors:** Rajiv Mahajan, Kapil Gupta

**Affiliations:** Department of Pharmacology, Adesh Institute of Medical Sciences and Research, Bathinda - 151 109, Punjab, India; 1 Department of Biochemistry, Adesh Institute of Medical Sciences and Research, Bathinda - 151 109, Punjab, India

**Keywords:** Adaptation, clinical trials, innovations, statistical analysis

## Abstract

New drug development is a time-consuming and expensive process. Recently, there has been stagnation in the development of novel compounds. Moreover, the attrition rate in clinical research is also on the rise. Fearing more stagnation, the Food and Drug Administration released the critical path initiative in 2004 and critical path opportunity list in 2006 thus highlighting the need of advancing innovative trial designs. One of the innovations suggested was the adaptive designed clinical trials, a method promoting introduction of pre-specified modifications in the design or statistical procedures of an on-going trial depending on the data generated from the concerned trial thus making a trial more flexible. The adaptive design trials are proposed to boost clinical research by cutting on the cost and time factor. Although the concept of adaptive designed clinical trials is round-the-corner for the last 40 years, there is still lack of uniformity and understanding on this issue. This review highlights important adaptive designed methodologies besides covering the regulatory positions on this issue.

## Introduction

Developing a new medicine is an expensive and time-consuming process. In the past several decades, it is recognized that increasing spending of biomedical research does not reflect an increase of the success rate of pharmaceutical development. The low success rate of pharmaceutical development could be due to the rapidly escalating costs and complexity leading to decreased willingness/ability to bring new candidates forward into the clinic.[1] Stagnation in the development of innovative products even gave an alarming call to the United States Food and Drug Administration (FDA). In a 2004 white paper, now known as the Critical Path Initiative (CPI), the FDA called attention to an alarming decline in the number of innovative medical products being submitted for FDA approval.[2] Unacceptable levels of attrition in the clinical stage of development are driving profound changes in the architecture, design and analysis of clinical trials. Moreover, the pharmaceutical industry is gradually coming to realize that the classically structured clinical trial does not offer enough flexibility to make use of continuously emerging knowledge that is generated as the trial progresses.[3] To bridge the gap between basic scientific research and medical product development, improved and innovative testing methods were drafted so as to ultimately improve the manner in which drugs are discovered, developed and brought into the market.[4]

One of the innovations strongly recommended by the FDA is the use of adaptive design methods in clinical trials and the potential use of the Bayesian approach in clinical research and development.[5] The European Medicines Agency (EMEA) has also issued a draft paper in 2006 concerning flexible or adaptive design clinical trials in new drug development.[6] The purpose of adaptation in clinical trials is to give the investigator the flexibility for identifying the optimal clinical benefit of the test treatment under study without undermining the validity and integrity of the intended study.[7] Although the concept of adaptive design clinical trials is still in its infancy but, with growing knowledge, positive signals have emerged from regulatory agencies and from industry alike.

## What is Adaptive Design Clinical Trial

An adaptive design is defined as a design that allows modifications to the trial and/or statistical procedures of the trial after its initiation without undermining its validity and integrity.[8] The purpose is to make clinical trials more flexible, efficient and fast. Due to the level of flexibility involved, these trial designs are also termed as “flexible designs.”

Flexibility here does not mean that the trial can be modified any time at will. The modification and adaptations have to be pre-planned and should be based on data collected from the study itself. Accordingly, the new draft guidance of the FDA for industry on adaptive design clinical trials defines an adaptive design clinical trial as “a study that includes a prospectively planned opportunity for modification of one or more specified aspects of the study design and hypotheses based on analysis of data (usually interim data) from subjects in the study.” Analyses of the accumulating study data are performed at pre-planned timepoints within the study, with or without formal statistical hypothesis testing.[9]

The term prospective here means that the adaptation was planned before data were examined in an unblinded manner. Changes in the study design occurring after an interim analysis of unblinded study data and those that were not prospectively planned are not within the scope of this guidance. Moreover, study design aspects that are revised based on information obtained entirely from sources outside of the specific study are not considered adaptive design, irrespective of the fact whether such adaptations were planned prospectively or occurred as a response to unanticipated external events. However, prospective study revisions based on information obtained from both a study-external and a study-internal source are considered adaptive designs.[9]

An adaptation is referred to a change made to the trial procedure and/or statistical procedure during the conduct of a clinical trial. Trial procedures may be the eligibility criteria, study dose, treatment duration, study endpoints, laboratory testing procedures, diagnostic procedures, criteria for evaluation and assessment of clinical responses. Statistical procedures include randomization, study design, study hypotheses, sample size, data monitoring and interim analysis, statistical analysis plan and/or methods for data analysis.[8]

## Background

The use of adaptive design methods for modifying the trial and/or statistical procedures of ongoing clinical trials based on accrued data has been practiced for years in clinical research. The concept of adaptive design can be traced back to the 1970s, when the adaptive randomization procedures and a class of designs for sequential clinical trials were introduced.[10] Due to this historical link-up, most adaptive design methods in clinical research are referred to as adaptive randomization,[11] group sequential designs[12] and sample size re-estimation at interim for achieving the desired statistical power.[13] In 1990, an improved Phase I design – the Continuous Re-Assessment Method (CRM) – was introduced, which gave a better control of the amount of toxicity observed during the trial, was flexible in the target level of maximum tolerated toxicity and gave a more reliable estimate of the maximum tolerated dose. This has given rise to a whole family of CRM designs.[14]

The potential use of adaptive design methods in clinical trials has been acknowledged recently. Working groups has been established for designing adaptive designs by the Pharmaceutical Research and Manufacturers of America (PhRMA) and Biotechnology Industry Organization (BIO).[15]

## Types of Adaptive Design Trials

Based on adaptations employed, commonly considered adaptive design methods in clinical trials include an adaptive randomization design, a group sequential design, a sample size re-estimation design, a drop-the-loser design, an adaptive dose finding design, a biomarker-adaptive design, an adaptive treatment-switching design, a hypothesis-adaptive design, an adaptive seamless phase II/III trial design and a multiple adaptive design.[10]

Another way of classifying adaptive design clinical trials is by categorizing them under different rules, which are mostly four in number. Allocation rule defines how the subjects will be allocated to different arms in a trial and comprises response-adaptive randomization and covariate adaptive allocation. Sampling rule defines how many subjects will be sampled at the next stage and consists of sample size re-estimation design (both blinded and unblinded) and drop-the-loser design. Stopping rule defines when to stop the trial and consists of group sequential design and adaptive treatment-switching design. Decision rule comprises changes not covered under the other three categories and consists of hypothesis-adaptive design and change the primary end-point or statistical method or patient population design. Sometimes, a fifth rule is added consisting of multiple adaptations, also comprising adaptive seamless phase II/III trial designs.[16,17]

### Adaptive randomization design

In adaptive randomization design alterations in the randomization schedule is allowed depending upon the varied or unequal probabilities of treatment assignment. The purpose is to increase the probability of success.[10] Commonly applied adaptive randomization procedures include treatment-adaptive randomization, covariate-adaptive randomization[18] and response-adaptive randomization.[11,19] It is not possible to use this adaptation in a trial of long duration as the proposed modification depends upon the response of the subjects already enrolled in the trial and as such trial will be more delayed.[10]

### Group sequential design

In group sequential design, a trial can be stopped prematurely if there are safety or efficacy issues and depending upon the results of the interim analysis, additional modifications can be made.[10] It should be noted that the standard methods for group sequential design may not be appropriate, like it may not be able to control the overall type I error rate at the desired level of 5%, if there is a shift in the target patient population due to additional adaptations or protocol amendments.[20,21]

Group sequential designs are already in use in the clinical oncology setup. The most familiar example is the “3+3” Phase I trial design for finding a maximum-tolerated-dose. In a 3+3 trial, three patients start at a given dose and, if no dose-limiting toxic effects are seen, three more patients are added to the trial at a higher dose. If there is one instance of limiting toxicity in the first group, three more patients are added at the same dose. If two (or all three) in any cohort show dose-limiting toxicity, the next lower dose is declared to be the maximum tolerated.[22]

### Sample size re-estimation design

A sample size can be modified or re-estimated in this type of design based on the observed data at interim, which can be performed in either a blinding or unblinding fashion based on the criteria of treatment effect-size, conditional power and/or reproducibility probability. One should not start with a small number of subjects initially and then do a sample size re-estimation at interim analysis, least one may miss the clinically meaningful difference of the ongoing trial.[10] It should be noted that the observed difference at interim analysis based on a small number of subjects may not be of statistical significance. Thus, standard methods for sample size re-estimation based on the observed difference with a limited number of subjects may be biased and misleading.[23,24]

### Drop-the-loser design

In this design the subjects detected to have received inferior treatments at the interim analysis can be dropped out. Based on the findings of the interim analysis, additional treatment arms can also be added at this stage.[10] This design is useful in Phase II clinical development, especially when there are uncertainties regarding the dose levels. Typically, drop-the-loser design is a two-stage design. At the end of the first stage, the inferior arms will be dropped based on some pre-specified criteria. The winners will then proceed to the next stage.[25,26] Some prefer to term these designs as *pick-the-winner designs*.

### Adaptive dose-finding design [Figure 1]

An adaptive dose-finding design is often used in early-phase clinical development to identify the minimum effective dose and the maximum tolerable dose, which is used to determine the dose level for the next phase clinical trials.[27] For the adaptive dose-finding design, the method of CRM[28] in conjunction with the Bayesian approach is usually considered.[29] The Bayesian approach was developed specifically to deal with new data as they come in and to update the probabilities under investigation. Instead of determining the likelihood that a drug’s efficacy could have occurred by chance, a Bayesian trial will give a probability that the drug was effective.[22]

**Figure 1:**
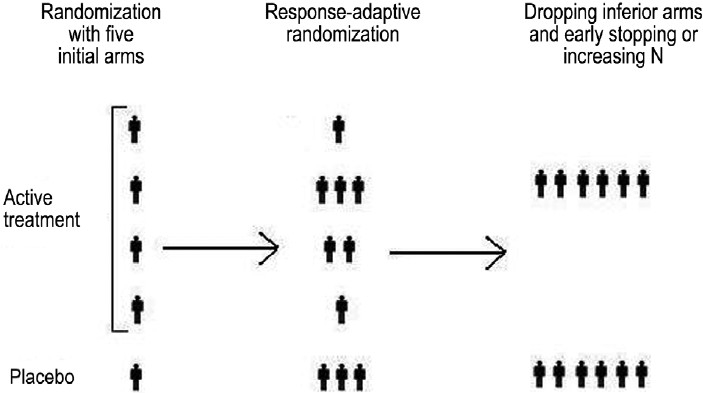
Drop-the-loser design (with intervening response-adaptive randomization)

### Biomarker-adaptive design

In this type of design, modification can be made in the ongoing trial based on the response of various biomarkers associated with the disease under consideration. A biomarker-adaptive design can be used to select the right patient population, identify natural course of disease, early detection of disease and to help in developing personalized medicine.[30,31] However, it should be kept in mind that there is a gap between identifying biomarkers associated with clinical outcomes and establishing a predictive model between relevant biomarkers and clinical outcomes in clinical development.[32]

### Adaptive treatment-switching design

In treatment-switching design, sifting the patient from one treatment option to other is allowed, if there are concerns about the safety or efficacy. But, in such type of trials estimation of survival rate will become very difficult, if the disease in question has poor prognosis. A high percentage of subjects may switch treatments due to disease progression, leading to confusion.[10]

In this case, sample size adjustment for achieving a desired power is necessary.[33]

### Hypothesis-adaptive design

An adaptive-hypotheses design refers to a design that allows modifications or changes in hypotheses based on interim analysis results. Adaptive-hypothesis design is often finalized before database lock or prior to data unblinding. Some examples include the switch from a superiority hypothesis to a non-inferiority hypothesis and the switch between the primary study endpoint and the secondary endpoints.[34]

### Adaptive seamless phase II/III design

This design combines the objectives of separate Phase IIb and Phase III in a single trial.[10] This design uses data from patients enrolled before and after the adaptation in the final analysis.[35] In a seamless-adaptive design, Phase II trial’s transitions into a Phase III trial happens without pause, saving considerable drug development time. Two sorts of seamless design are possible: operationally seamless, which simply seeks to exploit the saving in time; and inferentially seamless, which uses the new statistical methods to benefit from combining the relevant data from the Phase II part with the Phase III data in the final analysis.[14] An adaptive seamless phase II/III design is a two-stage design consisting of a so-called learning stage (Phase IIb) and a confirmatory stage (Phase III). A typical approach is to power the study for the Phase III confirmatory phase and to obtain valuable information with certain assurance using the confidence interval approach at the Phase II learning stage.[36]

Its validity and efficiency, however, have been challenged.[37] Moreover, it is not clear how to perform a combined analysis if the study objectives (or endpoints) are similar but different at different phases.[38] Although some modifications in test statistics and formulas for sample size calculation and allocation have been suggested for controlling the overall type I error at the pre-specified level, in cases in which the study objectives at different stages are different (e.g., dose finding at the first stage and efficacy confirmation at the second stage) and when there is a shift in patient population caused by protocol amendments, the picture is as yet far from satisfactory.[39]

### A multiple-adaptive design [Figure 2]

A multiple-adaptive design is any combination of the above adaptive designs. Commonly considered multiple-adaptation designs include either the combination of adaptive group sequential design, drop-the-loser design and adaptive seamless trial design or of adaptive dose-escalation design with adaptive randomization.[30] In practice, statistical inference for a multiple-adaptation design is often difficult.

**Figure 2:**
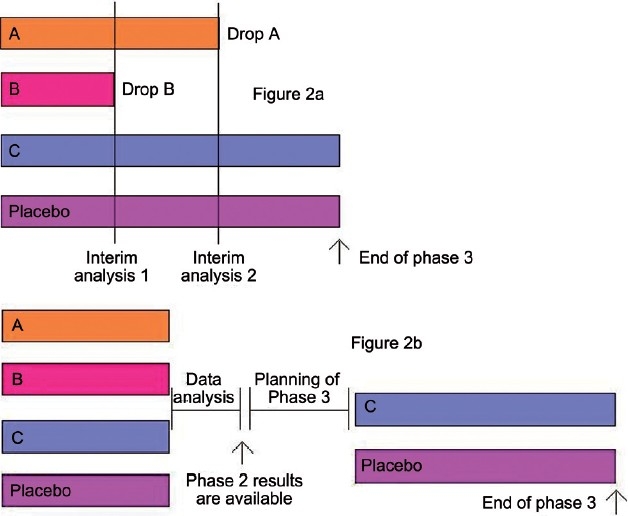
Adaptive seamless phase II/III design (2a) and its comparison with classical Phase II and III trials (2b)

## Challenges, Obstacles and Controversies in the Path of Adaptive Trials [Figure 3]

In practice, it is common to have three to five protocol amendments during the conduct of a clinical trial. One of the major impacts of many protocol amendments is that the target patient population may have been shifted during the process, which may have resulted in a totally different target patient population at the end of the trial. As a result, the resultant actual patient population following certain modifications to the trial procedures is a *moving* target patient population rather than a fixed target patient population and, consequently, the overall type I error rate may not be controlled.[40]

**Figure 3:**
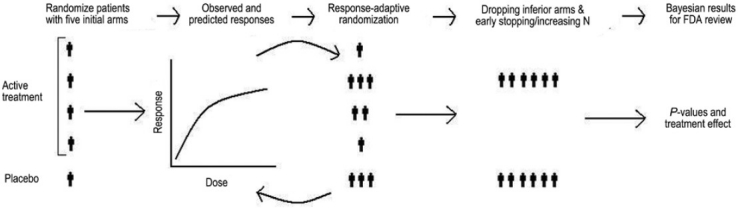
A multiple adaptive design (combining response-adaptive randomization, drop-the-loser design and seamless phase II/III design)[10]

In addition, major adaptations of trial and/or statistical procedures of ongoing trials may result in a totally different trial that is unable to address the clinical questions the trial intends to answer. Moreover, significant adaptations may introduce bias/variation to data collection as the trial continues.[40]

There are also some operational difficulties with adaptive trials.[41] These trials commonly use the Bayesian statistical approach, but this is computationally and logistically complex and might not be practically feasible in all situations. The sheer logistics of a high-level adaptive design also require careful thought. Quick and reliable electronic data collection would seem to be mandatory for a trial that is dependent on constant updating. To implement the adaptations, the data monitoring committee needs to meet regularly and quickly. Moreover, adaptive designs require computer-based simulations of clinical trials to develop the design and protocol, requiring more work force.[42] Adaptive trials will also make drug supply during the trial harder. Another area of concern is the current level of understanding of contact research organizations (CROs) about adaptive trials. Many CROs do not have long histories of carrying out these responsibilities. Therefore, study sponsors should have assurance that the personnel performing these studies on their behalf have appropriate expertise.[16]

There is also lack of uniformity on the definition of adaptive clinical trials. Moreover, it is only the FDA and, to some extent, the EMEA that seem to be proactive in implementing adaptive design clinical trials; other regulatory agencies are not so enthusiastic. There is also regulatory disagreement as there may be complete blurring of the exploratory and confirmatory phases in an adaptive trial.[43] Moreover, adaptations are encouraged in exploratory Phase I or IIa studies, but criticized in confirmatory Phase III studies. Few designs, like change the hypothesis or end-point or change in choice of test statistics, are controversial in themselves.[16]

## Regulatory Perspectives

Although flexibility of conduct of clinical trials associated with adaptive designs seem to be a very attractive prospect, the primary concern when implementing adaptive design methods in clinical trials is their validity and integrity from a regulatory perspective.[44] Thus, several regulatory concerns exist. First, what level of adaptation will be acceptable to the regulatory agencies? Second, what are the regulatory standards for the review and approval process of clinical data obtained from adaptive clinical trials with different levels of modifications? Third, has the clinical trial become a totally different clinical trial after the modifications for addressing the study objectives of the originally planned clinical trial?

These concerns should be addressed by the regulatory authorities before the adaptive design methods can be widely accepted in clinical research and development. Precisely, to address these concerns, the FDA released a draft guidance for industry on adaptive design clinical trials.[9] A similar draft was released by EMEA’s Committee for Medicinal Products for Human Use (CHMP) in 2006[6] and a joint workshop was organized by the US and EU regulators, academia and industry experts.[43]

Due to the increased complexity of some adaptive design studies and uncertainties regarding their performance characteristics, the FDA encourages earlier and more extensive interactions between the sponsor and the FDA than during a classical designed trial. During the early and middle periods of drug development following adaptive design clinical trials, sponsors having specific questions about the adaptive design elements in an exploratory study may seek FDA feedback either by identifying the specific issues and requesting a feedback along with protocol submission or by requesting a meeting to discuss specific questions.

During the later stages of drug development, if sufficient knowledge and information about a drug being developed is known, an adaptive design may be discussed initially at the End-of-Phase 2 meeting. However, if there is only limited knowledge of certain critical aspects of the drug’s use before conducting the adaptive study, discussion with the FDA earlier than usual is advisable, like at a End-of-Phase 2A meeting.[9]

The EMEA’s reflection paper on adaptive trials does not discuss specific statistical methods, but this paper highlighted the concern that while the increased flexibility that is now available may well fit the needs in early phases of drug development, their use in late Phase II or confirmatory Phase III trials deserves a more cautionary approach.[6] The joint US and EU regulator workshop also encouraged the use of adaptive designs in exploratory studies only and not in confirmatory studies.[45]

## Adaptive Design versus Conventional Trials

In conventional or classical clinical trials, one proceeds to accept/reject the null hypothesis in a well-defined population. No modification in trial design or statistical methods or patient’s population is permissible once a trial has started without documenting protocol amendments and the permission of the Institutional/Independent Ethics Committee, while in adaptive designs, pre-specified modifications are allowed based on the interim analysis. In classical trials, not more than two or three study arms are undertaken at a given time, while in adaptive design clinical trials, many treatment arms can be tested at the same time [Table 1].

**Table 1:** Comparison between conventional trial and adaptive design trial

*Features*	*Conventional trial*	*Adaptive design*
Design	More rigid	Flexible
Treatment arms	Maximum two or three	Many simultaneously
Hypothesis	Test the hypothesis under consideration	Fit data into a hypothesis
Modifications	Not allowed without protocol amendments	Pre-specified allowed
Phases	Phases I–II are well defined	Can be seamless phase 2/3 design
Statistical analysis	Use routine frequentists methods	Use complicated Bayesian approach
Organization	Much simple	Complicated, requiring simulations
Interim analysis	Not a routine	Done routinely and frequently
Role of IDMC	More once trial/phase is over	Proactive role throughout the trial
Regulatory view	Well endorsed	Still speculative

IDMC: Independent data-monitoring committee

### Advantages of adaptive designs

One obvious advantage of adaptive design trials is that potential modifications are approved before-hand by regulatory authorities and ethics committees and thus there is no need to file protocol amendments. Logistics for changing treatments or doses can also be planned upfront. Moreover, there is complete flexibility to react to unanticipated events and options exist to introduce any new doses, change endpoints etc. Credibility of results is also maintained, especially with blinded data or in the presence of firewalls, where only a limited number of people have access to the results.[46] There is also a broad regulatory acceptance, particularly in case of exploratory adaptive design clinical trials.[45]

Many compounds in drug development eventually fail, but these designs allow earlier detection and early stoppage of a clinical trial. Moreover, trial subjects are used more efficiently and fewer subjects are given ineffective compounds, ineffective doses or doses that are unnecessarily high. At the same time, pharmaceutical companies waste less on unsuccessful compounds and can more quickly re-assign resources to alternative drugs within their pipelines.

### Disadvantages and risks associated with adaptive designs

Implementing an adaptive design in drug development comes at a price. The first disadvantage is that after adaptation use of the Bayesian approach for statistical analyses is a compulsion rather than a choice, and many researchers still consider the Bayesian statistical methods as non-standard.[47] Even after applying the Bayesian statistical methods, sometimes, it become difficult to control the type 1 error in adaptive trials. Another risk is that ad hoc changes based on unblinded data may jeopardize the credibility of the study. Even EMEA’s draft has highlighted the possibility of damaging the integrity of a trial due to frequent interim analyses.[6] There is also the risk of development of a tendency of conducting adapting trials too early, thereby jeopardising the overall study findings. Above all, blanket regulatory acceptance is still far from sight.[46]

### How to minimize risk

To minimize the risk associated with frequent interim analyses, two important issues have been underlined to be considered by the EMEA draft of 2006. The first is that the need to perform any interim analysis should be very demanding and the second is that the total number of interim analyses should be well justified. Both these issues should be carefully planned and dealt with. A balance has to be achieved between the needs for assessing accumulating information and the risk of damaging the integrity of the trial. Routinely breaking the blind should be avoided, particularly when it can be foreseen that insufficient information will be available for stopping the study because of proven efficacy or futility or meaningful safety concerns of the experimental treatment.[6]

To further minimize the risk, changes to the design of an ongoing Phase III trial are not recommended. It is also recommended that studies with adaptive designs need at least the same careful investigation of heterogeneity and justification to combine the results of different stages as is usually required for the combination of individual trials in a metaanalysis.[6]

## Current Scenario

Current signals from the pharmaceutical industry are positive. Regulatory agencies in the major markets have developed decisive positions on adaptive design clinical trials. Many software development and information technology companies are developing software and clinical trial management system solutions to support adaptive design clinical trials.[48] Two-stage adaptive proof-of-concept and dose-finding adaptive designed clinical trials have already been used for migraine studies.[49] A 3+3 adaptive design is already in use in oncology studies.[22]

Recently, the “Biomarkers Consortium” launched a pioneering multi-agent adaptive clinical trial to treat breast cancer, intended to give several investigational drugs to treat breast cancer together at the same time, under a project named “Investigation of Serial Studies to Predict Your Therapeutic Response with Imaging And Molecular Analysis (I-SPY 2 TRIAL).” The Biomarkers Consortium is a unique public–private partnership led by the Foundation for the National Institutes of Health (NIH). In this trial, adaptive design will enable researchers to use early data from one set of patients to make decisions about which treatments might be more useful for patients later in the trial, and eliminate ineffective treatments more quickly.[50,51]

The Council of Scientific and Industrial Research of India has started an open-source drug discovery (OSDD) initiative, associating adaptive designs with pre-competitive research collaborations. This project is focused on targeting tuberculosis and is an internet-based project with no intellectual property.[52] The cause of adaptive designed clinical trials is certainly going to be boosted with the launch of adaptive designed clinical trials on such a large scale.

## Future Prospects

As indicated earlier, the use of adaptive design methods in clinical trials is very attractive due to its flexibility and efficiency for identifying best clinical benefits of the treatment under investigation. Not surprisingly, the new wave of adaptive clinical trials have sprung from these developments, driven by the growing difficulty of developing new drugs and enabled by the increasingly early availability of clinical data resulting from electronic data capture. Future prospects of these adaptive designed clinical trials are encouraging for both industry and regulatory agencies as well as from the patients’ perspective.

### For industry

These designs would allow more doses to be tested in Phases I and II, leading to a better understanding of the effect of the compound on patients in the doses that are clinically relevant thus leading to better decisions concerning the drug’s development and, for successful compounds, a better design of the Phase III trial, thereby helping to reduce the failure of a drug at this stage or, worse, later in its development. A seamless Phase I and Phase IIa trial would allow efficacy and toxicity to be studied at the outset, with the safety study directly in patients and not in healthy volunteers. This seamlessness would either allow the trial to be stopped early if the compound is ineffective or allow it to be continued with additional dose arms. It will also become possible to respond to changes in the market, like approval of a competitor product, and the overall effect will be that the drug development process will become less time consuming and cheaper.

### For regulatory agencies

These designs encourage a greater association between regulatory agencies and the sponsors from the outset of a trial thus minimizing chances of failure. They will be very handy for clinical development of drugs for rare diseases. With a more proactive role for regulatory agencies; more constructive collaboration between regulatory agencies, industry and academia in the form of consortiums or OSDD finding treatment options for difficult-to-treat conditions will be accelerated.

### For patients

These would minimize exposure to potentially harmful and unefficacious experimental treatments and would improve the understanding of the disease process, e.g. change visit schedules or dosing regimens.

## Conclusion

Rewards of adaptive designed clinical trials are many. These not only have a positive-predictive value, in the sense that efficacy of a drug is established earlier by using seamless designs, but also have a negative-predictive value, in the sense that ineffective treatments can be eliminated at an earlier stage. Regulatory agencies have also taken positive positions on adaptive trials. But, at this stage, caution is advised as drastic changes are often considered as lack of planning and can be met with criticism, especially when performed in late Phase II or Phase III trials for efficacy. Also, not every trial can be rescued by adaptation and adaptive designs. These should not be a cure for poor planning. Although, presently, there might be some problems in the execution of adaptive designs, with the release of draft guidance for industry on adaptive design clinical trials, more and more companies are bound to use adaptive designed clinical trials thus making the drug development process shorter and cheaper.
